# Crystal structure of (1*S*,2*R*,4*S*)-1-[(morpholin-4-yl)meth­yl]-4-(prop-1-en-2-yl)cyclo­hexane-1,2-diol

**DOI:** 10.1107/S2056989014027169

**Published:** 2015-01-01

**Authors:** Rachid Outouch, Saadia Oubaassine, Mustapha Ait Ali, Larbi El Firdoussi, Anke Spannenberg

**Affiliations:** a Laboratoire de Chimie de Coordination et de Catalyse, Département de Chimie, Faculté des Sciences Semlalia, BP 2390, 40001 Marrakech, Morocco; b Leibniz-Institut für Katalyse e. V. an der Universität Rostock, Albert-Einstein-Strasse 29a, 18059 Rostock, Germany

**Keywords:** crystal structure, hydrogen bonds, amino-1,2-diol, chiral ligand for catalytic enanti­oselective transformations

## Abstract

The asymmetric unit of the title compound, C_14_H_25_NO_3_, contains two independent mol­ecules with similar geometry. The morpholine and cyclo­hexane rings of both mol­ecules adopt a chair conformation. Intra­molecular O—H⋯N hydrogen bonds are observed. In the crystal, mol­ecules are linked by O—H⋯O hydrogen bonds into chains parallel to the [101] direction. The chains are further connected through C—H⋯O hydrogen bonds forming undulating layers parallel to the (-101) plane. The absolute configuration was assigned by reference to an unchanging chiral centre in the synthetic procedure.

## Abstract 2

Besides intra­molecular O—H⋯N hydrogen bonds, the crystal structure displays inter­molecular O—H⋯O and C—H⋯O hydrogen bonds linking the mol­ecules into undulating layers parallel to the (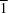
01) plane.

## Chemical context   

1,2-Amino­alcohols are important building blocks in the synthesis of natural products, pharmaceuticals and other materials (Möller, 1957 ▸). The classical synthetic approach towards amino­alcohols involves amino­lysis of epoxides with an excess of amines. There are some limitations to this classical approach, such as the requirement of elevated reaction temperatures in the case of less reactive amines, lower reactivity for sterically crowded amines/epoxides, and poor regioselectivity of the epoxide ring opening (Sello *et al.*, 2006 ▸). To obviate these problems, various methodologies to undertake epoxide opening under milder conditions have been developed (Surendra *et al.*, 2005 ▸), but there are still many limitations, such as the formation of bis­alkyl­ated products, longer reaction times, stoichiometric amounts of catalysts and harsh reaction conditions.

Recently, we have shown that calcium(II) compounds are very useful, environmentally friendly catalysts for several acid-catalysed reactions (Harrad *et al.*, 2010 ▸). Moreover, calcium triflate works under almost neutral conditions. In a continuation of our ongoing program on the amino­lysis of 1,2-epoxides using a mild, practical and efficient method under solvent-free conditions (Outouch, Boualy, Ali *et al.*, 2011 ▸; Outouch, Boualy, El Firdoussi *et al.*, 2011 ▸; Outouch *et al.*, 2014 ▸), we report herein the synthesis and crystal structure of a new amino­diol from ep­oxy­perillyl alcohol, which can be used as a chiral ligand for catalytic enanti­oselective transformations. The title compound was prepared by condensation of ep­oxy­perillyl alcohol with morpholine using a catalytic amount of Ca(CF_3_COO)_2_ under solvent-free conditions according to the procedure described previously (Outouch, Boualy, Ali *et al.*, 2011 ▸; Outouch, Boualy, El Firdoussi *et al.*, 2011 ▸).
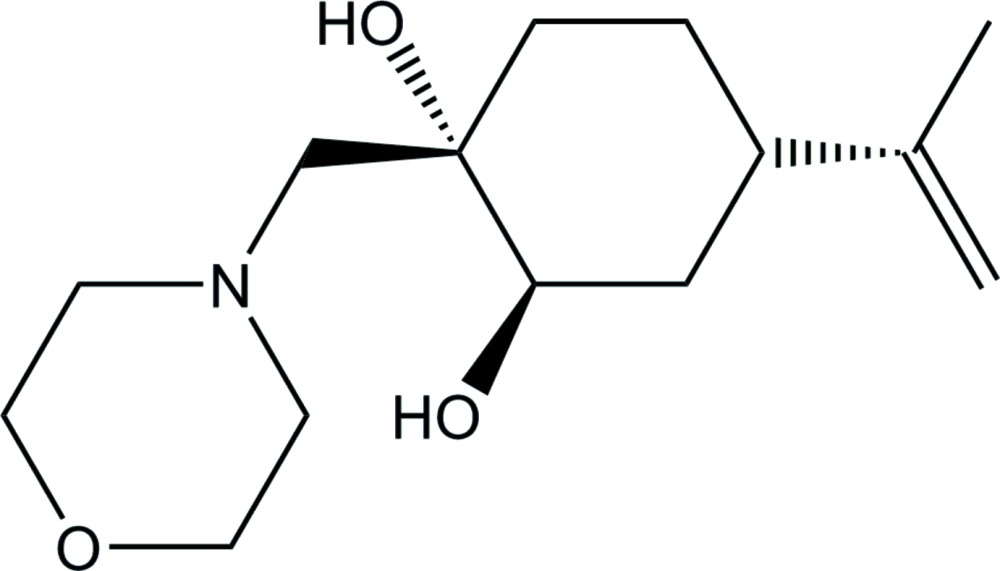


## Structural commentary   

**Figure 1:**
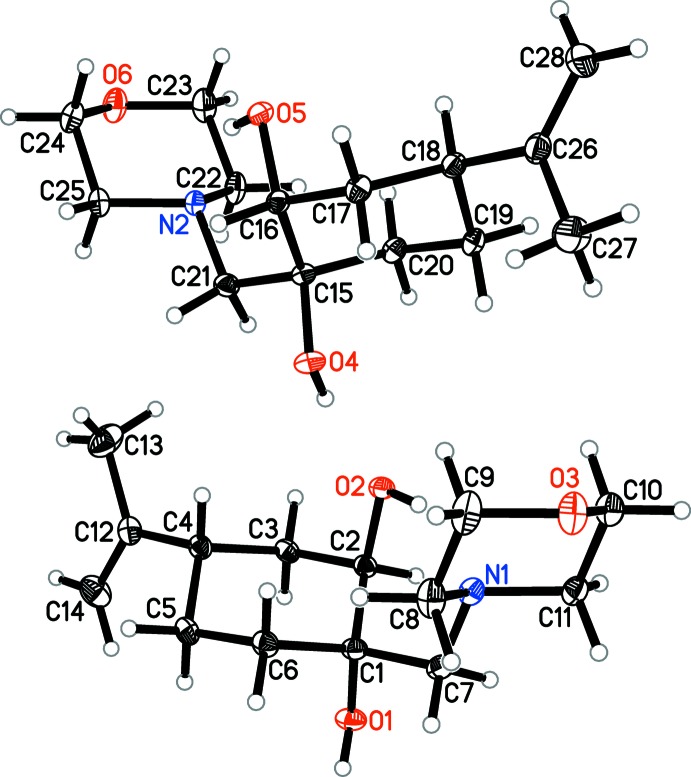
The mol­ecular structure of the two independent molecules of the title compound, with displacement ellipsoids drawn at the 30% probability level.

As shown in Fig. 1 ▸, there are two mol­ecules in the asymmetric unit of the title compound. In both mol­ecules, the cyclo­hexane rings adopt a chair conformation, with atoms C1/C4 and C15/C18 as flaps. The hydroxyl groups are all in axial positions. A chair conformation is also observed for the morpholine rings, with the N and O atoms as flaps. The mol­ecular conformation is enforced by an intra­molecular O—H⋯N hydrogen bond (Table 1 ▸).

## Supra­molecular features   

**Figure 2:**
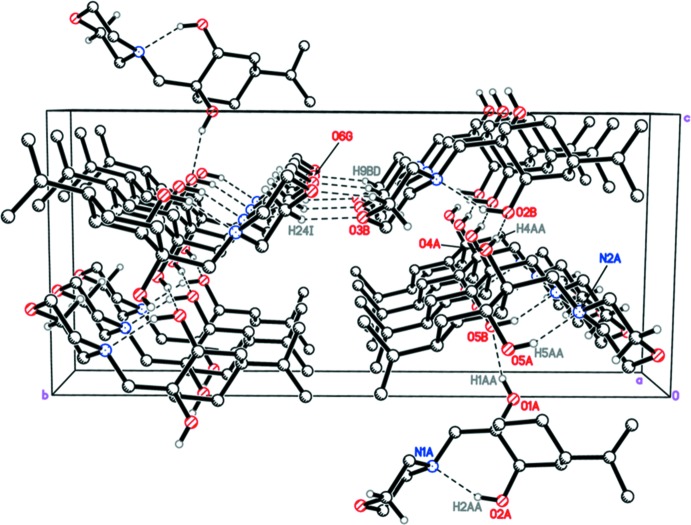
A packing diagram of the title compound showing hydrogen bonds as dashed lines (see Table 1 ▸ for details).

In the crystal, mol­ecules are linked by O—H⋯O hydrogen bonds (Table 1 ▸) involving the hydroxyl groups into chains running parallel to the [101] direction (Fig. 2 ▸). Moreover, the chains are further connected *via* C—H⋯O hydrogen bonds, forming undulating layers parallel to the (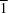
01) plane.

## Database survey   

The structures of related 1,4-substituted cyclo­hexane-1,2-diols have been reported recently by Byrne *et al.* (2004 ▸), Blair *et al.* (2007 ▸, 2010 ▸), Dams *et al.* (2004 ▸), Outouch, Boualy, Ali *et al.* (2011 ▸) and Outouch, Boualy, El Firdoussi *et al.* (2011 ▸). As found for the title compound, the cyclo­hexane-1,2-diol rings of these compounds adopt a chair conformation.

## Synthesis and crystallization   

A mixture of morpholine (5.1 mmol) and ep­oxy­perillyl alcohol (5 mmol), prepared by epoxidation of (*S*)-(−) perillyl alcohol, was added to 5 mol% of Ca(CF_3_CO_2_)_2_ under solvent-free conditions. The mixture was stirred at 313 K for 72 h. After the reaction had finished, the mixture was extracted with ethyl acetate (3 × 10 ml), dried over Na_2_SO_4_ and the solvent was removed at reduced pressure. The title compound was purified by column chromatography on silica gel using hexa­ne/ethyl acetate (1:1 *v*/*v*) as eluent (yield 49%). Single crystals suitable for X-ray analysis were obtained by slow evaporation of the solvents.

^1^H NMR (CDCl_3_): δ [p.p.m.] 1.8 (*s*, 3H), 2.3 (*m*, 1H), 2.59 (*m*, 2H), 2.66 (*s*, 2H), 3.31 (*m*, 1H), 3.67 (*m*, 4H), 4.68 (*s*, 2H); ^13^CNMR (CDCl_3_) δ [p.p.m.] 16.9, 21.4, 27.1, 29.4, 32, 51, 62.5, 63.4, 65.9, 67.9, 104.6, 144.9.

## Refinement   

Crystal data, data collection and structure refinement details are summarized in Table 2 ▸. The H atoms attached to oxygen could be found in a difference Fourier map and were freely refined. All other H atoms were placed in idealized positions with *d*(C—H) = 0.95–0.99 Å and refined using a riding model, with *U*_iso_(H) = 1.2 *U*_eq_(C) or 1.5 *U*_eq_(C) for methyl H atoms. A rotating model was adopted for the methyl groups. The absolute configuration was not established by anomalous scattering effects, the enanti­omer was assigned by reference to an unchanging chiral center in the synthetic procedure.

## Supplementary Material

Crystal structure: contains datablock(s) I. DOI: 10.1107/S2056989014027169/rz5144sup1.cif

Structure factors: contains datablock(s) I. DOI: 10.1107/S2056989014027169/rz5144Isup2.hkl

Supporting information file. DOI: 10.1107/S2056989014027169/rz5144Isup3.cml

CCDC reference: 1038806

Additional supporting information:  crystallographic information; 3D view; checkCIF report

## Figures and Tables

**Table 1:** Hydrogen-bond geometry (, )

*D*H*A*	*D*H	H*A*	*D* *A*	*D*H*A*
O2H2*A*N1	0.86(2)	1.91(2)	2.7118(19)	154(2)
O5H5*A*N2	0.83(3)	1.90(3)	2.6697(18)	155(3)
O1H1*A*O5^i^	0.84(3)	1.95(3)	2.7595(18)	164(3)
O4H4*A*O2	0.84(3)	2.00(3)	2.8249(17)	167(2)
C9H9*B*O6^ii^	0.99	2.35	3.269(2)	155
C24H24*A*O3^iii^	0.99	2.45	3.344(2)	150

Symmetry codes: (i) 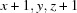; (ii) 
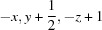
; (iii) 
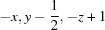
.

**Table 2:** Experimental details

Crystal data	
Chemical formula	C_14_H_25_NO_3_
*M*_r_	255.35
Crystal system, space group	Monoclinic, *P*2_1_
Temperature (K)	150
*a*, *b*, *c* ()	6.3300(1), 22.0241(5), 10.1179(2)
()	95.2083(12)
*V* (^3^)	1404.74(5)
*Z*	4
Radiation type	Mo *K*
(mm^1^)	0.08
Crystal size (mm)	0.44 0.42 0.28

Data collection	
Diffractometer	Bruker Kappa APEXII DUO
Absorption correction	Multi-scan (*SADABS*; Bruker, 2008 ▸)
*T*_min_, *T*_max_	0.92, 0.98
No. of measured, independent and observed [*I* > 2(*I*)] reflections	42775, 6792, 6514
*R*_int_	0.027
(sin /)_max_ (^1^)	0.660

Refinement	
*R*[*F* ^2^ > 2(*F* ^2^)], *wR*(*F* ^2^), *S*	0.031, 0.082, 1.06
No. of reflections	6792
No. of parameters	343
No. of restraints	1
H-atom treatment	H atoms treated by a mixture of independent and constrained refinement
_max_, _min_ (e ^3^)	0.24, 0.15
Absolute structure	Flack *x* determined using 3097 quotients [(*I* ^+^)(*I* )]/[(*I* ^+^)+(*I* )] (Parsons *et al.*, 2013 ▸)
Absolute structure parameter	0.21(19)

Computer programs: *APEX2* (Bruker, 2011 ▸), *SAINT* (Bruker, 2009 ▸), *SHELXS97*, *SHELXL2014* and *SHELXTL* (Sheldrick, 2008 ▸).

## supplementary crystallographic information

### Crystal data

**Table:**

C_14_H_25_NO_3_	*F*(000) = 560
*M**_r_* = 255.35	*D*_x_ = 1.207 Mg m^−^^3^
Monoclinic, *P*2_1_	Mo *K*α radiation, λ = 0.71073 Å
*a* = 6.3300 (1) Å	Cell parameters from 9947 reflections
*b* = 22.0241 (5) Å	θ = 2.7–28.8°
*c* = 10.1179 (2) Å	µ = 0.08 mm^−^^1^
β = 95.2083 (12)°	*T* = 150 K
*V* = 1404.74 (5) Å^3^	Prism, colourless
*Z* = 4	0.44 × 0.42 × 0.28 mm

### Data collection

**Table:**

Bruker Kappa APEXII DUO diffractometer	6792 independent reflections
Radiation source: fine-focus sealed tube	6514 reflections with *I* > 2σ(*I*)
Curved graphite monochromator	*R*_int_ = 0.027
Detector resolution: 8.3333 pixels mm^-1^	θ_max_ = 28.0°, θ_min_ = 1.9°
φ and ω scans	*h* = −8→7
Absorption correction: multi-scan (*SADABS*; Bruker, 2008)	*k* = −29→29
*T*_min_ = 0.92, *T*_max_ = 0.98	*l* = −13→13
42775 measured reflections	

### Refinement

**Table:**

Refinement on *F*^2^	Hydrogen site location: mixed
Least-squares matrix: full	H atoms treated by a mixture of independent and constrained refinement
*R*[*F*^2^ > 2σ(*F*^2^)] = 0.031	*w* = 1/[σ^2^(*F*_o_^2^) + (0.0479*P*)^2^ + 0.1776*P*] where *P* = (*F*_o_^2^ + 2*F*_c_^2^)/3
*wR*(*F*^2^) = 0.082	(Δ/σ)_max_ < 0.001
*S* = 1.06	Δρ_max_ = 0.24 e Å^−^^3^
6792 reflections	Δρ_min_ = −0.15 e Å^−^^3^
343 parameters	Absolute structure: Flack *x* determined using 3097 quotients [(*I*^+^)-(*I*^-^)]/[(*I*^+^)+(*I*^-^)] (Parsons *et al.*, 2013)
1 restraint	Absolute structure parameter: 0.21 (19)

### Special details

**Table:**

Geometry. All e.s.d.'s (except the e.s.d. in the dihedral angle between two l.s. planes) are estimated using the full covariance matrix. The cell e.s.d.'s are taken into account individually in the estimation of e.s.d.'s in distances, angles and torsion angles; correlations between e.s.d.'s in cell parameters are only used when they are defined by crystal symmetry. An approximate (isotropic) treatment of cell e.s.d.'s is used for estimating e.s.d.'s involving l.s. planes.

### Fractional atomic coordinates and isotropic or equivalent isotropic displacement parameters (Å^2^)

**Table:**

	*x*	*y*	*z*	*U*_iso_*/*U*_eq_	
C1	0.7333 (2)	0.28883 (8)	0.90710 (15)	0.0222 (3)	
C2	0.7379 (2)	0.25014 (8)	0.78061 (15)	0.0212 (3)	
H2B	0.8889	0.2453	0.7609	0.025*	
C3	0.6442 (3)	0.18737 (8)	0.79751 (16)	0.0231 (3)	
H3A	0.7398	0.1640	0.8616	0.028*	
H3B	0.6369	0.1658	0.7113	0.028*	
C4	0.4205 (3)	0.18895 (8)	0.84677 (16)	0.0228 (3)	
H4B	0.3248	0.2105	0.7780	0.027*	
C5	0.4259 (3)	0.22604 (8)	0.97478 (17)	0.0258 (3)	
H5B	0.2813	0.2285	1.0044	0.031*	
H5C	0.5187	0.2057	1.0454	0.031*	
C6	0.5091 (3)	0.28994 (8)	0.95188 (17)	0.0249 (3)	
H6A	0.5096	0.3136	1.0352	0.030*	
H6B	0.4128	0.3106	0.8835	0.030*	
C7	0.8332 (3)	0.35171 (8)	0.88857 (18)	0.0268 (4)	
H7A	0.8264	0.3750	0.9718	0.032*	
H7B	0.9850	0.3458	0.8756	0.032*	
C8	0.5642 (3)	0.42779 (8)	0.8175 (2)	0.0333 (4)	
H8A	0.6201	0.4555	0.8895	0.040*	
H8B	0.4525	0.4023	0.8517	0.040*	
C9	0.4707 (3)	0.46446 (8)	0.7005 (2)	0.0359 (4)	
H9A	0.4120	0.4367	0.6296	0.043*	
H9B	0.3531	0.4898	0.7280	0.043*	
C10	0.7961 (3)	0.46608 (8)	0.61057 (19)	0.0291 (4)	
H10A	0.9052	0.4926	0.5766	0.035*	
H10B	0.7409	0.4390	0.5374	0.035*	
C11	0.8970 (3)	0.42814 (8)	0.72373 (18)	0.0267 (3)	
H11A	1.0109	0.4027	0.6918	0.032*	
H11B	0.9615	0.4551	0.7946	0.032*	
C12	0.3362 (3)	0.12475 (8)	0.85890 (19)	0.0278 (3)	
C13	0.2009 (4)	0.10122 (10)	0.7415 (3)	0.0477 (6)	
H13A	0.1600	0.0592	0.7580	0.072*	
H13B	0.2808	0.1027	0.6629	0.072*	
H13C	0.0733	0.1264	0.7263	0.072*	
C14	0.3850 (4)	0.08997 (9)	0.9646 (2)	0.0404 (5)	
H14A	0.3336	0.0495	0.9662	0.048*	
H14B	0.4712	0.1056	1.0386	0.048*	
C15	0.1605 (2)	0.29160 (7)	0.44014 (15)	0.0190 (3)	
C16	−0.0367 (2)	0.32633 (7)	0.37967 (15)	0.0188 (3)	
H16	−0.1437	0.3279	0.4465	0.023*	
C17	0.0177 (2)	0.39080 (7)	0.34182 (16)	0.0219 (3)	
H17A	0.0615	0.4141	0.4235	0.026*	
H17B	−0.1106	0.4104	0.2978	0.026*	
C18	0.1962 (2)	0.39340 (7)	0.24867 (16)	0.0216 (3)	
H18	0.1467	0.3708	0.1659	0.026*	
C19	0.3920 (2)	0.36029 (7)	0.31378 (17)	0.0221 (3)	
H19A	0.5057	0.3606	0.2527	0.026*	
H19B	0.4453	0.3818	0.3960	0.026*	
C20	0.3380 (2)	0.29471 (7)	0.34709 (16)	0.0213 (3)	
H20A	0.4662	0.2745	0.3897	0.026*	
H20B	0.2932	0.2726	0.2641	0.026*	
C21	0.1026 (3)	0.22662 (7)	0.48039 (16)	0.0233 (3)	
H21A	0.2351	0.2051	0.5115	0.028*	
H21B	0.0150	0.2293	0.5564	0.028*	
C22	0.1256 (3)	0.14898 (8)	0.30774 (19)	0.0280 (4)	
H22A	0.1939	0.1195	0.3720	0.034*	
H22B	0.2385	0.1728	0.2703	0.034*	
C23	−0.0033 (3)	0.11532 (9)	0.1975 (2)	0.0344 (4)	
H23A	−0.0649	0.1448	0.1309	0.041*	
H23B	0.0907	0.0876	0.1528	0.041*	
C24	−0.3040 (3)	0.12046 (8)	0.3148 (2)	0.0295 (4)	
H24A	−0.4185	0.0963	0.3498	0.035*	
H24B	−0.3706	0.1503	0.2507	0.035*	
C25	−0.1826 (3)	0.15376 (8)	0.42746 (17)	0.0245 (3)	
H25A	−0.2796	0.1808	0.4715	0.029*	
H25B	−0.1206	0.1243	0.4939	0.029*	
C26	0.2417 (3)	0.45830 (8)	0.20956 (19)	0.0277 (4)	
C27	0.3227 (4)	0.50158 (9)	0.3166 (2)	0.0448 (5)	
H27A	0.3413	0.5418	0.2780	0.067*	
H27B	0.4592	0.4870	0.3582	0.067*	
H27C	0.2206	0.5042	0.3836	0.067*	
C28	0.2082 (4)	0.47606 (11)	0.0848 (2)	0.0479 (6)	
H28A	0.2356	0.5169	0.0616	0.057*	
H28B	0.1568	0.4480	0.0182	0.057*	
N1	0.7366 (2)	0.38889 (7)	0.77770 (14)	0.0231 (3)	
N2	−0.0127 (2)	0.18982 (6)	0.37589 (13)	0.0201 (3)	
O1	0.8751 (2)	0.25829 (6)	1.00335 (13)	0.0293 (3)	
O2	0.62372 (19)	0.27966 (6)	0.66952 (11)	0.0238 (2)	
O3	0.6273 (2)	0.50262 (6)	0.65029 (16)	0.0343 (3)	
O4	0.2236 (2)	0.32243 (6)	0.56239 (12)	0.0256 (3)	
O5	−0.12856 (18)	0.29577 (6)	0.26338 (11)	0.0218 (2)	
O6	−0.1697 (2)	0.08119 (6)	0.24837 (15)	0.0340 (3)	
H1A	0.876 (4)	0.2765 (12)	1.076 (3)	0.041 (7)*	
H2A	0.657 (4)	0.3175 (11)	0.678 (2)	0.028 (5)*	
H4A	0.334 (4)	0.3044 (11)	0.593 (3)	0.037 (6)*	
H5A	−0.121 (4)	0.2593 (14)	0.282 (3)	0.046 (7)*	

### Atomic displacement parameters (Å^2^)

**Table:**

	*U*^11^	*U*^22^	*U*^33^	*U*^12^	*U*^13^	*U*^23^
C1	0.0204 (7)	0.0277 (8)	0.0183 (7)	0.0030 (6)	−0.0002 (5)	−0.0015 (6)
C2	0.0194 (7)	0.0260 (8)	0.0185 (7)	0.0024 (6)	0.0031 (6)	0.0000 (6)
C3	0.0257 (7)	0.0241 (8)	0.0201 (7)	0.0035 (6)	0.0052 (6)	−0.0001 (6)
C4	0.0222 (7)	0.0243 (8)	0.0222 (7)	0.0020 (6)	0.0031 (6)	0.0032 (6)
C5	0.0256 (8)	0.0288 (8)	0.0243 (8)	0.0026 (6)	0.0089 (6)	0.0011 (6)
C6	0.0251 (7)	0.0272 (8)	0.0233 (7)	0.0033 (6)	0.0067 (6)	−0.0032 (6)
C7	0.0244 (8)	0.0298 (9)	0.0255 (8)	−0.0022 (6)	−0.0018 (6)	−0.0032 (6)
C8	0.0315 (9)	0.0240 (8)	0.0468 (11)	0.0021 (7)	0.0166 (8)	−0.0044 (8)
C9	0.0263 (9)	0.0216 (8)	0.0612 (13)	0.0044 (7)	0.0115 (8)	0.0018 (8)
C10	0.0266 (8)	0.0264 (8)	0.0351 (9)	0.0023 (6)	0.0082 (7)	−0.0019 (7)
C11	0.0207 (7)	0.0285 (8)	0.0311 (9)	−0.0024 (6)	0.0030 (6)	−0.0033 (7)
C12	0.0259 (8)	0.0263 (8)	0.0318 (9)	0.0007 (6)	0.0061 (7)	0.0037 (7)
C13	0.0461 (12)	0.0315 (10)	0.0621 (15)	−0.0090 (9)	−0.0141 (11)	0.0070 (10)
C14	0.0572 (13)	0.0283 (9)	0.0367 (10)	0.0008 (9)	0.0094 (9)	0.0068 (8)
C15	0.0187 (6)	0.0196 (7)	0.0183 (7)	0.0005 (6)	−0.0005 (5)	−0.0021 (6)
C16	0.0162 (6)	0.0213 (7)	0.0191 (7)	0.0008 (5)	0.0020 (5)	−0.0009 (5)
C17	0.0182 (7)	0.0202 (7)	0.0273 (8)	0.0024 (6)	0.0017 (6)	−0.0007 (6)
C18	0.0203 (7)	0.0213 (7)	0.0229 (7)	−0.0022 (6)	0.0005 (6)	−0.0007 (6)
C19	0.0160 (6)	0.0225 (7)	0.0280 (8)	−0.0013 (5)	0.0033 (6)	−0.0021 (6)
C20	0.0167 (7)	0.0206 (7)	0.0267 (8)	0.0020 (6)	0.0023 (5)	−0.0030 (6)
C21	0.0260 (8)	0.0226 (8)	0.0209 (8)	−0.0003 (6)	−0.0006 (6)	0.0029 (6)
C22	0.0224 (7)	0.0227 (8)	0.0406 (10)	−0.0017 (6)	0.0127 (7)	−0.0030 (7)
C23	0.0385 (10)	0.0277 (9)	0.0397 (10)	−0.0078 (7)	0.0172 (8)	−0.0096 (8)
C24	0.0216 (7)	0.0253 (8)	0.0421 (10)	−0.0036 (6)	0.0052 (7)	−0.0026 (7)
C25	0.0225 (7)	0.0229 (7)	0.0291 (8)	−0.0008 (6)	0.0083 (6)	0.0027 (6)
C26	0.0241 (8)	0.0252 (8)	0.0334 (9)	−0.0041 (6)	0.0008 (7)	0.0035 (7)
C27	0.0647 (15)	0.0237 (9)	0.0440 (12)	−0.0115 (9)	−0.0061 (10)	0.0017 (8)
C28	0.0576 (14)	0.0441 (12)	0.0398 (12)	−0.0206 (10)	−0.0071 (10)	0.0147 (9)
N1	0.0197 (6)	0.0220 (6)	0.0277 (7)	0.0012 (5)	0.0034 (5)	−0.0034 (5)
N2	0.0195 (6)	0.0186 (6)	0.0225 (6)	−0.0013 (5)	0.0040 (5)	−0.0002 (5)
O1	0.0291 (6)	0.0371 (7)	0.0207 (6)	0.0061 (5)	−0.0041 (5)	0.0004 (5)
O2	0.0275 (6)	0.0245 (6)	0.0188 (5)	−0.0021 (4)	−0.0013 (4)	0.0011 (4)
O3	0.0307 (7)	0.0207 (6)	0.0533 (9)	0.0033 (5)	0.0136 (6)	0.0011 (6)
O4	0.0268 (6)	0.0272 (6)	0.0218 (6)	0.0015 (5)	−0.0047 (5)	−0.0056 (5)
O5	0.0203 (5)	0.0220 (6)	0.0223 (6)	−0.0013 (4)	−0.0032 (4)	−0.0001 (5)
O6	0.0328 (7)	0.0229 (6)	0.0478 (8)	−0.0077 (5)	0.0124 (6)	−0.0082 (6)

### Geometric parameters (Å, º)

**Table:**

C1—O1	1.4308 (19)	C15—C21	1.541 (2)
C1—C6	1.529 (2)	C15—C16	1.543 (2)
C1—C2	1.540 (2)	C16—O5	1.4321 (18)
C1—C7	1.541 (2)	C16—C17	1.518 (2)
C2—O2	1.4355 (19)	C16—H16	1.0000
C2—C3	1.520 (2)	C17—C18	1.537 (2)
C2—H2B	1.0000	C17—H17A	0.9900
C3—C4	1.543 (2)	C17—H17B	0.9900
C3—H3A	0.9900	C18—C26	1.518 (2)
C3—H3B	0.9900	C18—C19	1.534 (2)
C4—C12	1.520 (2)	C18—H18	1.0000
C4—C5	1.529 (2)	C19—C20	1.529 (2)
C4—H4B	1.0000	C19—H19A	0.9900
C5—C6	1.528 (2)	C19—H19B	0.9900
C5—H5B	0.9900	C20—H20A	0.9900
C5—H5C	0.9900	C20—H20B	0.9900
C6—H6A	0.9900	C21—N2	1.472 (2)
C6—H6B	0.9900	C21—H21A	0.9900
C7—N1	1.476 (2)	C21—H21B	0.9900
C7—H7A	0.9900	C22—N2	1.470 (2)
C7—H7B	0.9900	C22—C23	1.515 (3)
C8—N1	1.472 (2)	C22—H22A	0.9900
C8—C9	1.509 (3)	C22—H22B	0.9900
C8—H8A	0.9900	C23—O6	1.428 (2)
C8—H8B	0.9900	C23—H23A	0.9900
C9—O3	1.428 (2)	C23—H23B	0.9900
C9—H9A	0.9900	C24—O6	1.424 (2)
C9—H9B	0.9900	C24—C25	1.506 (2)
C10—O3	1.424 (2)	C24—H24A	0.9900
C10—C11	1.511 (3)	C24—H24B	0.9900
C10—H10A	0.9900	C25—N2	1.470 (2)
C10—H10B	0.9900	C25—H25A	0.9900
C11—N1	1.476 (2)	C25—H25B	0.9900
C11—H11A	0.9900	C26—C28	1.320 (3)
C11—H11B	0.9900	C26—C27	1.498 (3)
C12—C14	1.329 (3)	C27—H27A	0.9800
C12—C13	1.493 (3)	C27—H27B	0.9800
C13—H13A	0.9800	C27—H27C	0.9800
C13—H13B	0.9800	C28—H28A	0.9500
C13—H13C	0.9800	C28—H28B	0.9500
C14—H14A	0.9500	O1—H1A	0.84 (3)
C14—H14B	0.9500	O2—H2A	0.86 (2)
C15—O4	1.4354 (18)	O4—H4A	0.84 (3)
C15—C20	1.531 (2)	O5—H5A	0.83 (3)
			
O1—C1—C6	110.33 (13)	O5—C16—C17	108.46 (12)
O1—C1—C2	104.34 (13)	O5—C16—C15	110.28 (12)
C6—C1—C2	110.06 (13)	C17—C16—C15	111.69 (12)
O1—C1—C7	105.45 (13)	O5—C16—H16	108.8
C6—C1—C7	115.08 (14)	C17—C16—H16	108.8
C2—C1—C7	110.94 (13)	C15—C16—H16	108.8
O2—C2—C3	109.03 (13)	C16—C17—C18	112.70 (13)
O2—C2—C1	110.74 (13)	C16—C17—H17A	109.1
C3—C2—C1	111.89 (13)	C18—C17—H17A	109.1
O2—C2—H2B	108.4	C16—C17—H17B	109.1
C3—C2—H2B	108.4	C18—C17—H17B	109.1
C1—C2—H2B	108.4	H17A—C17—H17B	107.8
C2—C3—C4	113.24 (13)	C26—C18—C19	113.25 (13)
C2—C3—H3A	108.9	C26—C18—C17	111.25 (14)
C4—C3—H3A	108.9	C19—C18—C17	109.22 (13)
C2—C3—H3B	108.9	C26—C18—H18	107.6
C4—C3—H3B	108.9	C19—C18—H18	107.6
H3A—C3—H3B	107.7	C17—C18—H18	107.6
C12—C4—C5	114.17 (14)	C20—C19—C18	110.82 (12)
C12—C4—C3	110.14 (13)	C20—C19—H19A	109.5
C5—C4—C3	109.58 (14)	C18—C19—H19A	109.5
C12—C4—H4B	107.6	C20—C19—H19B	109.5
C5—C4—H4B	107.6	C18—C19—H19B	109.5
C3—C4—H4B	107.6	H19A—C19—H19B	108.1
C6—C5—C4	110.19 (13)	C19—C20—C15	111.65 (13)
C6—C5—H5B	109.6	C19—C20—H20A	109.3
C4—C5—H5B	109.6	C15—C20—H20A	109.3
C6—C5—H5C	109.6	C19—C20—H20B	109.3
C4—C5—H5C	109.6	C15—C20—H20B	109.3
H5B—C5—H5C	108.1	H20A—C20—H20B	108.0
C5—C6—C1	111.87 (13)	N2—C21—C15	115.84 (13)
C5—C6—H6A	109.2	N2—C21—H21A	108.3
C1—C6—H6A	109.2	C15—C21—H21A	108.3
C5—C6—H6B	109.2	N2—C21—H21B	108.3
C1—C6—H6B	109.2	C15—C21—H21B	108.3
H6A—C6—H6B	107.9	H21A—C21—H21B	107.4
N1—C7—C1	116.42 (13)	N2—C22—C23	109.96 (14)
N1—C7—H7A	108.2	N2—C22—H22A	109.7
C1—C7—H7A	108.2	C23—C22—H22A	109.7
N1—C7—H7B	108.2	N2—C22—H22B	109.7
C1—C7—H7B	108.2	C23—C22—H22B	109.7
H7A—C7—H7B	107.3	H22A—C22—H22B	108.2
N1—C8—C9	110.15 (16)	O6—C23—C22	110.94 (15)
N1—C8—H8A	109.6	O6—C23—H23A	109.5
C9—C8—H8A	109.6	C22—C23—H23A	109.5
N1—C8—H8B	109.6	O6—C23—H23B	109.5
C9—C8—H8B	109.6	C22—C23—H23B	109.5
H8A—C8—H8B	108.1	H23A—C23—H23B	108.0
O3—C9—C8	111.02 (16)	O6—C24—C25	111.56 (14)
O3—C9—H9A	109.4	O6—C24—H24A	109.3
C8—C9—H9A	109.4	C25—C24—H24A	109.3
O3—C9—H9B	109.4	O6—C24—H24B	109.3
C8—C9—H9B	109.4	C25—C24—H24B	109.3
H9A—C9—H9B	108.0	H24A—C24—H24B	108.0
O3—C10—C11	112.01 (15)	N2—C25—C24	109.51 (14)
O3—C10—H10A	109.2	N2—C25—H25A	109.8
C11—C10—H10A	109.2	C24—C25—H25A	109.8
O3—C10—H10B	109.2	N2—C25—H25B	109.8
C11—C10—H10B	109.2	C24—C25—H25B	109.8
H10A—C10—H10B	107.9	H25A—C25—H25B	108.2
N1—C11—C10	110.28 (14)	C28—C26—C27	121.16 (18)
N1—C11—H11A	109.6	C28—C26—C18	120.70 (18)
C10—C11—H11A	109.6	C27—C26—C18	118.13 (16)
N1—C11—H11B	109.6	C26—C27—H27A	109.5
C10—C11—H11B	109.6	C26—C27—H27B	109.5
H11A—C11—H11B	108.1	H27A—C27—H27B	109.5
C14—C12—C13	120.99 (18)	C26—C27—H27C	109.5
C14—C12—C4	122.92 (18)	H27A—C27—H27C	109.5
C13—C12—C4	116.03 (16)	H27B—C27—H27C	109.5
C12—C13—H13A	109.5	C26—C28—H28A	120.0
C12—C13—H13B	109.5	C26—C28—H28B	120.0
H13A—C13—H13B	109.5	H28A—C28—H28B	120.0
C12—C13—H13C	109.5	C8—N1—C11	108.20 (14)
H13A—C13—H13C	109.5	C8—N1—C7	112.39 (14)
H13B—C13—H13C	109.5	C11—N1—C7	110.78 (13)
C12—C14—H14A	120.0	C22—N2—C25	108.76 (13)
C12—C14—H14B	120.0	C22—N2—C21	113.35 (13)
H14A—C14—H14B	120.0	C25—N2—C21	111.93 (13)
O4—C15—C20	110.29 (12)	C1—O1—H1A	109.2 (18)
O4—C15—C21	105.41 (12)	C2—O2—H2A	104.9 (15)
C20—C15—C21	114.16 (13)	C10—O3—C9	109.32 (13)
O4—C15—C16	105.01 (12)	C15—O4—H4A	104.7 (17)
C20—C15—C16	110.28 (13)	C16—O5—H5A	105.3 (19)
C21—C15—C16	111.20 (13)	C24—O6—C23	110.02 (13)
			
O1—C1—C2—O2	−172.39 (12)	C16—C17—C18—C26	178.24 (13)
C6—C1—C2—O2	69.23 (17)	C16—C17—C18—C19	−56.02 (17)
C7—C1—C2—O2	−59.30 (17)	C26—C18—C19—C20	−178.30 (14)
O1—C1—C2—C3	65.74 (16)	C17—C18—C19—C20	57.12 (17)
C6—C1—C2—C3	−52.63 (17)	C18—C19—C20—C15	−58.41 (17)
C7—C1—C2—C3	178.83 (12)	O4—C15—C20—C19	−60.07 (16)
O2—C2—C3—C4	−69.99 (16)	C21—C15—C20—C19	−178.51 (13)
C1—C2—C3—C4	52.85 (18)	C16—C15—C20—C19	55.46 (16)
C2—C3—C4—C12	179.02 (13)	O4—C15—C21—N2	166.67 (13)
C2—C3—C4—C5	−54.58 (17)	C20—C15—C21—N2	−72.14 (18)
C12—C4—C5—C6	−179.04 (14)	C16—C15—C21—N2	53.40 (18)
C3—C4—C5—C6	56.89 (17)	N2—C22—C23—O6	−58.5 (2)
C4—C5—C6—C1	−59.77 (18)	O6—C24—C25—N2	59.16 (19)
O1—C1—C6—C5	−57.93 (18)	C19—C18—C26—C28	120.7 (2)
C2—C1—C6—C5	56.68 (18)	C17—C18—C26—C28	−115.8 (2)
C7—C1—C6—C5	−177.09 (14)	C19—C18—C26—C27	−60.1 (2)
O1—C1—C7—N1	170.17 (14)	C17—C18—C26—C27	63.4 (2)
C6—C1—C7—N1	−67.99 (18)	C9—C8—N1—C11	57.64 (19)
C2—C1—C7—N1	57.78 (18)	C9—C8—N1—C7	−179.71 (14)
N1—C8—C9—O3	−60.3 (2)	C10—C11—N1—C8	−56.23 (18)
O3—C10—C11—N1	57.89 (18)	C10—C11—N1—C7	−179.86 (14)
C5—C4—C12—C14	−40.6 (2)	C1—C7—N1—C8	89.85 (18)
C3—C4—C12—C14	83.2 (2)	C1—C7—N1—C11	−148.98 (14)
C5—C4—C12—C13	142.11 (19)	C23—C22—N2—C25	57.74 (18)
C3—C4—C12—C13	−94.1 (2)	C23—C22—N2—C21	−177.07 (14)
O4—C15—C16—O5	−173.83 (12)	C24—C25—N2—C22	−57.80 (17)
C20—C15—C16—O5	67.37 (16)	C24—C25—N2—C21	176.19 (13)
C21—C15—C16—O5	−60.32 (16)	C15—C21—N2—C22	97.33 (17)
O4—C15—C16—C17	65.49 (16)	C15—C21—N2—C25	−139.20 (14)
C20—C15—C16—C17	−53.31 (17)	C11—C10—O3—C9	−58.3 (2)
C21—C15—C16—C17	179.00 (13)	C8—C9—O3—C10	59.2 (2)
O5—C16—C17—C18	−66.97 (15)	C25—C24—O6—C23	−58.9 (2)
C15—C16—C17—C18	54.76 (17)	C22—C23—O6—C24	58.2 (2)

### Hydrogen-bond geometry (Å, º)

**Table:**

*D*—H···*A*	*D*—H	H···*A*	*D*···*A*	*D*—H···*A*
O2—H2*A*···N1	0.86 (2)	1.91 (2)	2.7118 (19)	154 (2)
O5—H5*A*···N2	0.83 (3)	1.90 (3)	2.6697 (18)	155 (3)
O1—H1*A*···O5^i^	0.84 (3)	1.95 (3)	2.7595 (18)	164 (3)
O4—H4*A*···O2	0.84 (3)	2.00 (3)	2.8249 (17)	167 (2)
C9—H9*B*···O6^ii^	0.99	2.35	3.269 (2)	155
C24—H24*A*···O3^iii^	0.99	2.45	3.344 (2)	150

Symmetry codes: (i) *x*+1, *y*, *z*+1; (ii) −*x*, *y*+1/2, −*z*+1; (iii) −*x*, *y*−1/2, −*z*+1.
